# Balloon-Occluded Trans-Arterial Chemo-Embolization Technique with Repeated Alternate Infusion of Cisplatin Solution and Sparse Gelatin Slurry (RAIB-TACE) for Large Hepatocellular Carcinoma Nodules More than 7 cm in Diameter

**DOI:** 10.1155/2020/9289321

**Published:** 2020-01-19

**Authors:** Toshiyuki Irie, Nobuyuki Takahashi, Toshiro Kamoshida, Junya Kashimura, Hiroyuki Ariga

**Affiliations:** ^1^Department of Radiology, Tsukuba University Hospital Mito Clinical Education and Training Center, Mito Kyodo General Hospital, Mito, Japan; ^2^Department of Radiology, Tsukuba Memorial Hospital, Tsukuba, Japan; ^3^Department of Hepato-Gastroenterology, Hitachi General Hospital, Hitachi, Japan; ^4^Department of Gastroenterology, Tsukuba University Hospital Mito Clinical Education and Training Center, Mito Kyodo General Hospital, Mito, Japan

## Abstract

**Objective:**

It is sometimes difficult to obtain complete/partial response of large hepatocellular carcinoma (HCC) nodules by trans-arterial chemoembolization/embolization (TACE/TAE). The aim is retrospective investigation of tumor response of large HCC nodules (>7 cm) treated by the new TACE technique, repeated alternate infusion of cisplatin solution, and sparse gelatin slurry under balloon occlusion (RAIB-TACE). *Materials and Methods*. A microballoon catheter was placed at a proximal portion of the hepatic artery (subsegmental to the lobar level hepatic artery), and alternate infusion of cisplatin solution and sparse gelatin slurry were repeated under balloon occlusion until stasis of gelatin slurry beyond the catheter was seen. RAIB-TACE of multiple proximal hepatic and extrahepatic collateral arteries were performed to treat hemi-lobe or more of the liver while avoiding infusion into gastric and cystic arteries for 19 large nodules (>7 cm) in 19 patients without portal venous invasion. All patients underwent dynamic CT/MRI 1–3 months after RAIB-TACE, and tumor response of each large nodule was evaluated based on modified RECIST criteria.

**Results:**

CR, PR, SD, and PD were achieved in 11, 8, 0, and 0 nodules, respectively. CR and PR were considered as success, and the ratio of success was 100%. Major complications were abscess formation in the necrotic nodule (*n* = 1) which was treated by drainage tube placement, and subsegmental level liver infarction (*n* = 1) which was treated by drainage tube placement, and subsegmental level liver infarction (

**Conclusion:**

New TACE technique, RAIB-TACE, was useful to achieve successful response of large HCC nodules.

## 1. Introduction

Hepatocellular carcinoma (HCC) is one of the most common malignancies worldwide. The role of trans-arterial chemoembolization (TACE) is to treat HCC when neither surgery nor radiofrequency ablation (RFA) is indicated [1]. The HCC diameter is one of the main factors that determine TACE efficacy of the nodules [2]. With regard to the diameter criteria associated with a poor TACE efficacy, 4 cm was mentioned by some experts [3]. A surveillance study among Japanese experts showed that 5–7 cm was mentioned by 33% of experts, 7–10 cm by 36%, and ≥10 cm by 21% [4]. Thus, 7 cm is considered by approximately half of experts as the diameter criteria for TACE refractoriness.

It is well known that superselective lipiodol TACE for a small nodule via each peripheral tumor feeder is highly effective [5]. But it is time-consuming to apply this technique for a large nodule with many tumor feeders. It is also difficult to apply superselective lipiodol TACE technique for a small nodule adjacent to Glisson sheath because it is often supplied via multiple very small tumor feeders directly branching from a trunk artery. Simultaneous treatment of multiple tumor feeders could be done by nonselective lipiodol TACE technique via the proximal hepatic artery. However, nonselective lipiodol TACE is not preferred because it sometimes causes poor liver tolerance and less effective tumor control compared with selective lipiodol TACE [6, 7]. However, small nodules adjacent to Glisson sheath could be successfully treated by newly developed balloon-occluded TACE technique. Alternate infusion of cisplatin solution and sparse gelatin slurry was repeated via the trunk artery under balloon occlusion until stasis of gelatin slurry was seen beyond the catheter (RAIB-TACE) [8]. By using this technique, multiple tumor feeders could be treated simultaneously and effectively via a proximal portion of the hepatic artery. The concept of RAIB-TACE is the improvement of nonselective TACE technique to treat wide region (e.g., segmental level to the whole liver) by minimizing liver parenchymal damage and maximizing antitumor effect.

The purpose of this paper was retrospective investigation of the RAIB-TACE technique for the treatment of large HCC nodules more than 7 cm in diameter.

## 2. Materials and Methods

### 2.1. Characteristics of Patients and the Large Nodules (Table 1)

Between December 2014 and April 2018, a total of 19 large nodules (>7 cm) in 19 patients were treated by the RAIB-TACE technique. Eighteen patients had no past history of TACE nor RFA treatment. One patient had past history of 3 sessions of TACE, 2 sessions of superselective lipiodol B-TACE using doxorubicin (Adriacin injection, Kyowa Hakko, Tokyo) and mitomycin C (Mitomycin injection, Kyowa Hakko, Tokyo), and 1 session of RAIB-TACE for the whole liver. In this patient, a rapidly growing large HCC nodule mainly supplied via a renal capsular artery was treated. No patients were associated with portal/hepatic venous tumor thrombosis. Seven patients were with a solitary nodule, 4 with 3 nodules, and the rest 8 were with 10 or more nodules. Six patients with BCLC stage A were included, and these did not undergo surgery due to being elderly (*n* = 4, 85 years old or more), bi-lobar location of the large nodule (*n* = 1), and true rupture (*n* = 1). Nine nodules were more than 10 cm in diameter. The true rupture was seen in 2 and impending one in 4. We aimed to treat the entire large nodule and intrahepatic metastatic nodules (IHMNs) by a single session of RAIB-TACE. Second session of RAIB-TACE was performed 3 months or later after the first session of RAIB-TACE in 12 patients for treatment of remaining viable parts or recurred nodules.

### 2.2. Preparation of Gelatin Slurry

Dried spherical porous gelatin particles (GelpartⓇ, Nipponkayaku, Osaka) were commercially available while preserved in a glass bottle containing 80 mg of particles. Immediately before use, particles were mixed with nondiluted contrast medium (Oypalomin, Iopamidol 300, Fuji Pharma, Tokyo) to crush by pumping method between 2 small size syringes (2.5 mL in capacity) through a partially opened 3-way stopcock. The diameter of the original particle was 1 mm in wet state. The diameter of the tumor feeding artery at the entry site into the nodule was often less than 1 mm [9], and we considered that the use of original size particles would cause proximal embolization. Thus, gelatin slurry containing smaller fragments (136.8 ± 60.8 to 261.4 ± 110.3 *μ*m, in wet state, 16 trials of crushing) were created to enhance antitumor effect by crushing the original size particles [10]. Gelatin slurry was diluted with 18 mL of contrast medium, and this sparse gelatin slurry was charged in a medium size syringe (25 mL in capacity) which was connected with another medium size syringe using a fully opened 3-way stopcock. Immediately before injection, sparse gelatin slurry was stirred by pumping method to prevent aggregation of fragments, and 1 mL of stirred sparse gelatin slurry was transferred into a small size syringe (1 mL in capacity).

### 2.3. The RAIB-TACE Technique

**Figure 1:**
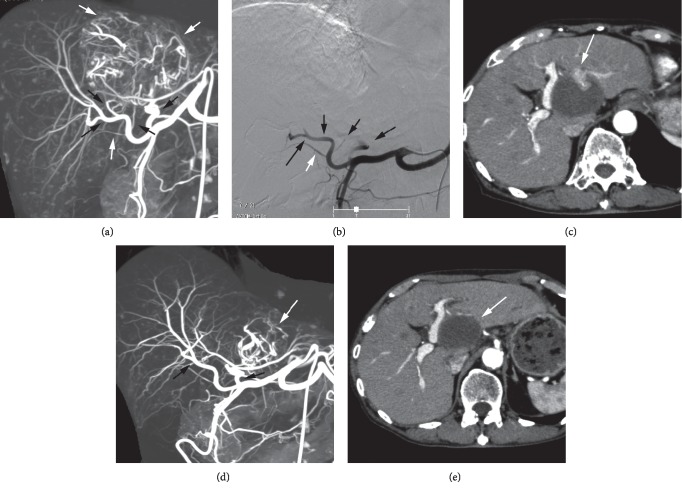
RAIB-TACE for a large nodule in the liver hilum. Immediately before the first session of RAIB-TACE, MIP image of trans-arterial MDCT angiography via the celiac artery well depicted hepatic arterial branches and the large nodule (large white arrow, A) in the liver hilum. Multiple tiny high density spots were arterioportal shunts. RAIB-TACE was performed for right hepatic (large black arrow, A), medial segmental (small black arrow, A), caudate segmental (small black arrowhead, A), and left hepatic arteries (large black arrowhead, A). Post-TACE DSA showed occlusion of all hepatic arterial branches (black arrows and arrowheads, B) and patent cystic artery (white arrowhead, B). CT 3 months after RAIB-TACE depicted a remaining viable part (white arrow, C), and PR was achieved. The second session of RAIB-TACE was performed 5 months after the first one. MIP image of trans-arterial MDCT angiography via the celiac artery showed patent hepatic arterial branches (black arrow, black arrowhead, D) and tumor vessels in a remaining viable part of the large nodule (white arrow, D). RAIB-TACE could be successfully done via the left hepatic artery (black arrowhead, D). CR of the large nodule (white arrow, E) was achieved after the second session of RAIB-TACE.

For all patients, trans-arterial multidetector rows CT (MDCT) angiography (MDCTA) via celiac (*n* = 14), superior mesenteric (*n* = 4), or common trunk artery (*n* = 1) was performed to obtain 3 dimensional (3D) data set of hepatic arterial system (Figure 1). The patient was transferred to the CT room after catheter placement. Nondiluted contrast medium was infused at a rate of 5 mL/sec for 8–10 seconds and scan was performed 4–5 seconds after the start of injection using 64-MDCT (Scenaria, Hitachi Medico, Kashiwa) or 80-MDCT (Aquilion prime SP 80, Canon, Tokyo). The appropriate catheter positions for TACE were decided based on this 3D data set. The tip of a microballoon catheter (Attendant LP/nexus, Terumo, Tokyo, and Logos, Piolax, Yokohama) was placed at the proximal hepatic artery (subsegmental to the lobar level artery) while avoiding infusion into cystic, right gastric, and accessory left gastric arteries. Extrahepatic collateral arteries were detected by referring arterial phase CT/MRI image and also treated by the RAIB-TACE technique.

Fine cisplatin powder (IA coal, Nippon Kayaku, Osaka) was solved with saline as 1 mg/mL. This cisplatin solution and sparse gelatin slurry were alternately and rapidly infused into the artery under balloon occlusion and repeated 1–9 times. Approximately 25 mg of cisplatin was used for a segment of liver, and a total of 50–112 mg (mean: 77 mg) was used for each patient. The endpoint of TACE was the filling of sparse gelatin slurry in arteries like tree structure beyond the balloon catheter and its stasis after balloon deflation. When the vascular lake was seen during TACE procedures, gelatin cubes 1.5–2 mm in length were prepared by cutting a gelatin block using scissors. Infusion of 1–3 cubes was repeated 1–3 times under balloon deflation until the vascular lake disappeared.

Systemic hydration was performed to prevent cisplatin renal disturbance immediately after a patient was transferred into the angiographic room. Rapid hydration of 1000 mL/2-3 hours followed by drip infusion of 1000 mL/12 hours was done.

### 2.4. Analysis

All 19 patients underwent dynamic CT or MRI 1–3 months after RAIB-TACE. The primary goal of this study was the analysis of tumor response of each large nodule on dynamic CT/MRI obtained 1–3 months after the first session of RAIB-TACE by applying modified RECIST criteria [11]. The results of the second session of RAIB-TACE for the remaining viable parts of the large nodule were also analyzed. Tumor response of intrahepatic metastatic nodules (IHMNs), complications, and causes of death were also analyzed.

CR and PR of the large nodule were considered as success. Adverse events on blood chemistry data after the first session of RAIB-TACE were assessed according to the National Cancer Institute Common Terminology Criteria (version 4.0). The analyzed factors were alanine aminotransferase (ALT) increase level and total bilirubin increase level. Severe adverse events such as tumor-lysis syndrome and acute liver failure, or major ones such as biloma, abscess, biliary stricture, and infarction were also evaluated.

**Figure 2:**
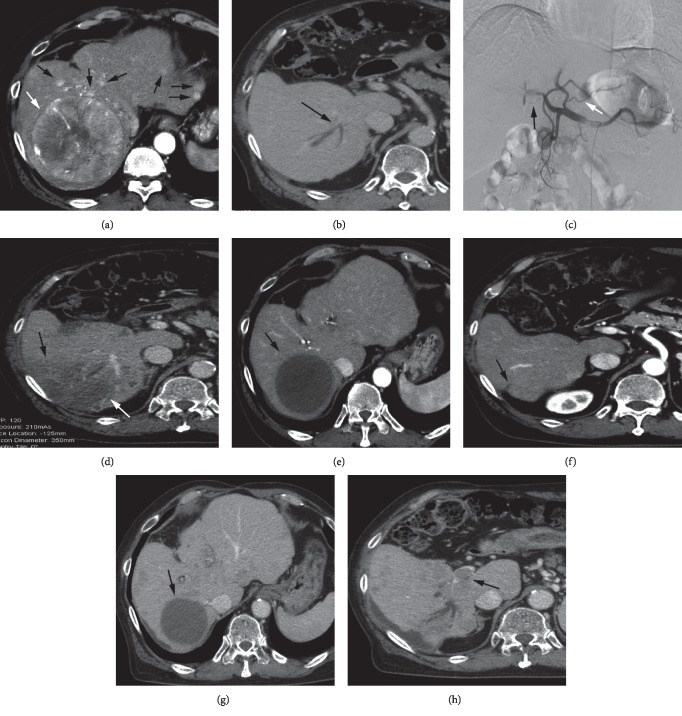
Subsegmental level liver infarction after RAIB-TACE. On arterial phase CT, a large nodule (white arrow, A) and multiple intrahepatic metastases (black arrows, A) were seen. On portal phase CT, dilatation of biliary tract in subsegment 7 (black arrow, B) was seen due to compression of proximal Glisson sheath by the large nodule. RAIB-TACE for the bi-lobe of the liver was done. Post-TACE DSA depicted patent cystic (black arrow, C) and right gastric arteries (white arrow, C) while occlusion of all hepatic arterial branches was achieved. Grade 4 increase of serum ALT level was seen on the next day after RAIB-TACE, and CT obtained 3 days after RAIB-TACE showed low density area (black arrows, D) that was compatible with liver infarction. On CT 3 months after RAIB-TACE, CR of the large nodule (black arrow, E) and disappearance of intrahepatic metastases were also shown. Dimpling of the liver surface (black arrow, F) was seen in the region of liver infarction. On CT 21 months after RAIB-TACE, CR of the large nodule was maintained (black arrow, G), but portal venous tumor thrombosis (black arrow, H) and rib metastases (not shown) were also seen.

Preservation of the hepatic artery after TACE is necessary to perform the next session of TACE. In RAIB-TACE, the endpoint of embolization was stasis of sparse gelatin slurry in the proximal portion of hepatic arteries (Figures 1 and 2). In DEB-TACE, the endpoint of embolization was reported as the disappearance of tumor stain [12]. Thus, the embolization degree of RAIB-TACE was different and so strong compared with DEB-TACE, and the influence of RAIB-TACE on patency of hepatic artery was investigated in patients referring to 3D data sets of hepatic arterial system and DSA when the second session of RAIB-TACE was performed.

### 2.5. Overall Survival

After dynamic CT for evaluation of tumor response, 3 were excluded from follow up due to being elderly (91 years old), Parkinson's disease, and Alzheimer's disease. RAIB-TACE was performed for these 3 patients to treat true rupture (*n* = 1) or impending rupture (*n* = 2). It was predetermined before RAIB-TACE that no additional treatments nor follow up by hepatologists would be done for these 3 patients and they were excluded from overall survival analysis. Overall survival was evaluated for the remaining 16 patients (Table 2) using the Kaplan–Meier method.

## 3. Results

### 3.1. The RAIB-TACE Procedures

The entire liver was treated for 6 patients and hemi-lobe or more for 13. Vascular lake [13] was seen in 17 of 19 large nodules and all were successfully managed. Eleven extrahepatic arteries were treated in 10 patients: right inferior phrenic artery (*n* = 6), left phrenic one (*n* = 1), capsular one (*n* = 3), and omental one (*n* = 1). In a patient with a ruptured nodule, rerupture occurred 6 hours after RAIB-TACE due to recanalization of a tumor feeder. This rerupture was treated successfully by superselective TAE of the tumor feeder.

### 3.2. Response of the Large Nodules and IHMNs

After the first session of RAIB-TACE, CR, PR, SD, and PD were achieved in 11, 8, 0, and 0 large nodules, respectively, and success ratio was 100% (Table 3). For 9 huge nodules 10 cm or more, CR and PR were achieved in 5 and 4, respectively (Table 3). CR, PR, SD, and PD of IHMNs were achieved in 3, 8, 1, and 0 patients, respectively (Figure 2). The second session of RAIB-TACE for the remaining viable parts of 5 large nodules was performed, and CR was achieved in 2 (40%) (Figure 1).

### 3.3. Adverse Events, Complications, and Patency of Hepatic Arteries

No severe complications such as death, liver failure, or tumor-lysis syndrome were seen. An abscess was seen in a completely necrotic large nodule in one patient with bilio-enteric anastomosis. A drainage tube was placed percutaneously in this abscess, and it was successfully treated. Subsegmental level liver infarction was seen in one, and the cause was compression of the proximal portion of subsegmental portal branch by the large nodule (Figure 2). This patient was recovered uneventfully, but shrinkage of the liver parenchyma occurred in the infarcted region.

Bilirubin increased level was grade 1–3 in all 19 patients and ALT increase one was grade 1–3 in 18, but grade 4 increase of ALT was seen in one who showed liver infarction described above (Table 4). These increased levels were returned to pretreatment levels one month after RAIB-TACE.

The second session of RAIB-TACE was performed in 12 patients 3 months or later after the first session of RAIB-TACE (Figure 1). No occlusion of hepatic arterial branches (subsubsegmental or more proximal level) was seen in any patients.

### 3.4. Overall Survival

**Figure 3:**
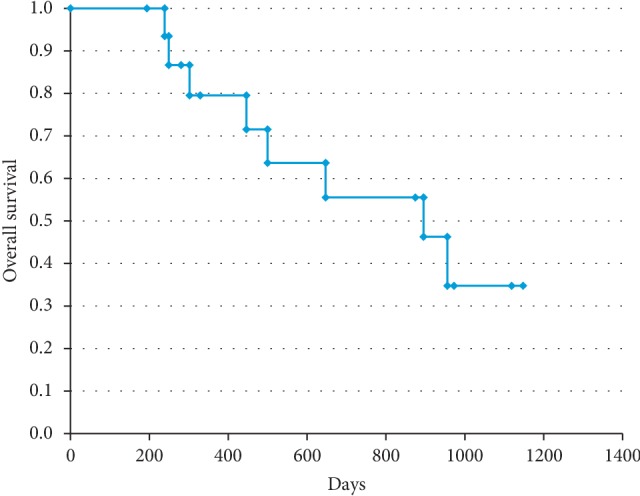
Kaplan–Meier survival curve. The overall survival was calculated using Kaplan–Meier method for 16 patients.

One was lost from follow up due to nonfatal cerebral bleeding 4 months after RAIB-TACE, and one due to refusal of follow up 6 months after RAIB-TACE. Eight patients died during the study period and the causes of death were deterioration of intrahepatic metastases or vascular invasion (Figure 2). In no case, enlargement or rupture of the large nodules caused death. The median survival duration was 877 days (Figure 3).

## 4. Discussion

In this retrospective study, CR could be achieved in 11 of 19 large nodules and PR in rest 8 nodules after the first session of RAIB-TACE. The objective response rate by RAIB-TACE was 100% and the CR ratio was 58%. Nine huge nodules more than 10 cm were also included, and either CR (*n* = 5) or PR (*n* = 4) could be achieved. A large-scale retrospective cohort study showed that lipiodol TACE could achieve objective response (CR or PR) for huge nodules more than 10 cm in diameter in 67 of 232 patients (28.9%) [14]. Recently, the use of drug eluting beads was recommended rather than lipiodol to treat large nodules 5 cm or more [15]. However, when the diameter exceeded 5 cm, CR could be achieved in 13 of 74 nodules (17.6%) [16]. RAIB-TACE was superior to these TACE techniques to treat large nodules.

Balloon occlusion prevented dilution of cisplatin by blood flow and enabled rapid infusion while preventing regurgitation of gelatin slurry. Rapid infusion of gelatin slurry prevented the aggregation of gelatin fragments in the catheter and proximal arteries. We considered crushing of 1 mm gelatin particles into smaller fragments and no aggregation of them as the key points for efficient cease of tumor blood flow. The use of 1 mm gelatin particles would cause proximal embolization and residual blood supply via collaterals such as peribiliary plexus, isolated artery, and communicating arcade [17–19]. The embolization of small materials from inside the nodule could exclude this collateral blood supply to the nodule. It was reported that the main tumor feeder diameter at the entry site into the nodule was often less than 1 mm [9].

However, as seen during TACE procedures using drug eluting beads, the vascular lake was frequently seen by using small gelatin fragments during the RAIB-TACE procedures (17/19, 89%). Seki et al. reported that the vascular lake indicated rupture of tumor vessels inside the nodule [13] and recommended treatment of the vascular lake. We also considered that the vascular lake should be embolized and eliminated because it could cause tumor rupture, and no safety evidence was available for its presence.

The second session of RAIB-TACE was attempted for 12 patients, no occlusion of hepatic arterial branches (subsubsegmental or more proximal level) was seen in any patients, and it could be successfully performed for all 12. We speculated that because gelatin slurry was sparsely prepared, RAIB-TACE did not cause occlusion of hepatic branches. In 5 of 12 patients, the aim was to treat the residual viable part of the large nodule, and it was effective to achieve CR in 2 of 5 (40%). We considered that the cause of the viable part remaining would be proximal embolization due to aggregation of gelatin fragments.

Before the development of the RAIB-TACE technique, we had applied selective B-TACE using lipiodol to treat large HCC nodules [20]. Selective B-TACE had an effect to increase the accumulation of lipiodol in nodules and improved local control rates, but superselective balloon catheter placement in each peripheral artery was necessary to obtain this effect [21]. Thus, it was time-consuming to treat a large nodule with many tumor feeding arteries. Moreover, when the diameter of the nodule exceeds 4 or 5 cm, it was impossible to achieve a dense accumulation of lipiodol because the lipiodol dose per patient was limited to 10 mL in our hospitals. Another disadvantage of lipiodol B-TACE was that intrahepatic arteries might be obscured by densely accumulated lipiodol in the large nodule after lipiodol was infused into the main tumor feeding arteries. On the contrary, during the RAIB-TACE procedures, it was easy to confirm sufficient filling of gelatin slurry in hepatic arteries beyond the catheter because the large nodule was slightly radiopaque with angiographic contrast medium even after TACE of the main tumor feeding arteries. It was also easy to identify extrahepatic tumor feeding arteries and stain of residual mass on DSA after RAIB-TACE of the main tumor feeding arteries. Thus, the cease of blood flow into the large nodule could be easily confirmed when RAIB-TACE was applied.

We experienced 2 major complications: abscess and subsegmental liver infarction. The cause of abscess was bilio-enteric anastomosis [22] and that of infarction was compression of the proximal portal branch. The possibility of both was predicted before RAIB-TACE, but we performed RAIB-TACE because we considered the merit of mass reduction would be superior to the demerits of these complications. No fatal complications such as tumor-lysis syndrome [23, 24] were seen in this study. But the risk should be taken into consideration when large HCCs were treated by TACE.

In this study, all large nodules could be successfully treated. Eight patients died during the study period and the causes of death were deterioration of intrahepatic metastases or progression of portal venous tumor thrombosis (Figure 2). Enlargement or rupture of the large nodule did not cause death in any patient. Thus, RAIB-TACE prevented death due to enlargement or rupture of the large nodule. Efficient treatment of intrahepatic metastases and portal venous thrombosis was necessary to improve the overall survival, for examples, by using molecular targeted agents [25].

There were several limitations to this study. The case population was small. Moreover, this study was a retrospective analysis. A prospective study was necessary.

In conclusion, RAIB-TACE achieved CR/PR of large hepatocellular carcinoma nodules more than 7 cm in diameter in all 19 nodules (100%). No severe complications such as death or liver failure were seen in any patients. RAIB-TACE prevented the large nodule to be the cause of death, but it was still unclear whether CR/PR of large nodule could prolong the overall survival. After the achievement of CR/PR of the large nodule, combined treatment such as administration of molecular targeted agent [25] would be useful to control intrahepatic metastases and to prevent portal venous tumor thrombosis for prolongation of the overall survival.

## Figures and Tables

**Table 1:** Characteristics of patients and the large nodules.

Male : female	14 : 5
Age (years)	56–91 (mean: 75.6)
True rupture : impending one	2 : 4
Causes of hepatitis (B : C : alcohol : NASH : PBC : unknown)	0 : 10 : 2 : 5 : 1 : 1
Child-Pugh stage (A : B)	16 : 3
Elevation of AFP (>100)^1^	8
Elevation of PIVKA-II (>100)^2^	11
No. of nodules (solitary : 3 : >10)	7 : 4 : 8
BCLC stage (A : B : C : D)^3^	6 : 11 : 0 : 2
Diameter of the large nodule (mm)	72–148 (mean: 99)
Dose of cisplatin (mg)	50–112 (mean: 79)

^1^AFP: alpha-fetoprotein. ^2^PIVKA-II: protein induced by vitamin K absence or antagonists-II. ^3^The cause of stage D was performance status 4 due to Parkinson's and Alzheimer's diseases. The Child-Pugh stage of both cases was B. The mean diameter of the large nodules was 99 mm.

**Table 2:** Follow up of patients.

19 Patients
↓
All 19 patients underwent dynamic CT or MRI 1–3 months after RAIB-TACE
↓
3 were not followed up thereafter due to being elderly (*n* = 1), Parkinson's disease (*n* = 1), and Alzheimer's disease (*n* = 1). These 3 were associated with true rupture (*n* = 1) or impending one (*n* = 2)
↓
2 were lost from follow up due to cerebral bleeding (*n* = 1) or refusal (*n* = 1).
↓
8 patients died due to deterioration of intrahepatic metastases and/or portal venous tumor thrombosis.

All 19 patients underwent follow-up CT/MRI for evaluation of tumor response.

**Table 3:** Responses of the large nodules and intrahepatic metastatic nodules (IHMNs).

CR : PR : SD : PD (all 19 nodules)	11 : 8 : 0 : 0^1^
CR : PR : SD : PD (9 huge ones > 10 cm)	5 : 4 : 0 : 0
CR : PR : SD : PD (IHMNs in 12 patients)	3 : 8 : 1 : 0

^1^CR of the large nodule was achieved in 2 of the 5 PR ones after the second session of RAIB-TACE. CR of the large nodules was achieved in 11 of 19 nodules (58%) after the first session of RAIB-TACE, and PR of the rest nodules. The objective response rate was 100%.

**Table 4:** Adverse events on blood chemistry data.

ALT increase level (grade 1 : 2 : 3 : 4)	6 : 3 : 9 : 1
Bilirubin increase level (grade 1 : 2 : 3 : 4)	7 : 9 : 3 : 0

ALT: Alanine aminotransferase. Adverse events were evaluated based on CTCAE (common terminology criteria for adverse events).

## Data Availability

The clinical data used to support the findings of this study have not been made available because no allowance of the review board of each hospital to disclose the data is obtained.
